# Biochemical Responses to Experimentally Induced Short‐Term Low Energy Availability in Athletes: A Systematic Review

**DOI:** 10.1111/sms.70249

**Published:** 2026-03-07

**Authors:** Isabel Guisado‐Cuadrado, Paula Recacha‐Ponce, Ana B. Peinado, Nuria Romero‐Parra

**Affiliations:** ^1^ LFE Research Group, Departamento de Salud y Rendimiento Humano Facultad de Ciencias de la Actividad Física y del Deporte (INEF), Universidad Politécnica de Madrid Madrid Spain; ^2^ Faculty of Health Sciences Jaime I University Castelló de la Plana Spain; ^3^ Department of Physical Therapy, Occupational Therapy, Rehabilitation, and Physical Medicine, Faculty of Health Sciences Universidad Rey Juan Carlos Madrid Spain

**Keywords:** bone remodeling, endocrine adaptations, energy deficiency, hormonal biomarkers, metabolic regulation

## Abstract

Low energy availability (LEA) occurs when dietary energy intake is insufficient to meet the combined demands of exercise and essential physiological functions. Problematic LEA is recognized as the primary driver of Relative Energy Deficiency in Sport (RED‐S), but the short‐term physiological consequences of LEA remain less clearly defined. Thus, this systematic review aimed to synthesize evidence from experimental studies examining the short‐term effects of experimentally induced LEA on biochemical markers in athletic populations. A systematic search was conducted in PubMed, Web of Science, and Scopus in accordance with PRISMA guidelines. Eligible studies included experimental designs, with pre–post assessments of an LEA intervention (> 24 h). Outcomes included biomarkers of bone metabolism, calcium metabolism, energy regulation, inflammation, iron status, sex hormones, and thyroid function. Thirteen studies (145 participants) were included. Approximately half of the interventions reported significant increases in βCTX‐1 and reductions in P1NP. Leptin consistently decreased following LEA, whereas IGF‐1 and T3 remained stable in most studies, and testosterone decreased in 50% of interventions. No consistent changes were observed in estradiol, progesterone, calcium metabolism, inflammatory markers, or iron status. Short‐term experimentally induced LEA elicits early endocrine and metabolic adaptations, particularly affecting bone remodeling, leptin, and testosterone. However, these responses should be interpreted in the context of the frequent coexistence of low carbohydrate availability, which may contribute to or exacerbate the observed effects. These findings emphasize the relevance of monitoring key biochemical markers during periods of potential LEA risk and underscore the need for standardized, sex‐specific protocols in future research.

## 1 Introduction

Energy Availability is defined as the amount of dietary energy remaining for essential physiological functions after accounting for the energy cost of exercise, typically expressed relative to fat‐free mass (kcal · kg FFM^−1^ · day^−1^) [1]. When energy availability falls, adaptive mechanisms are triggered to conserve energy, leading to the downregulation of reproductive, metabolic, and bone‐related processes [2]. Chronic low energy availability (LEA) is recognized as the primary underlying mechanism of Relative Energy Deficiency in Sport (RED‐S), a clinical syndrome encompassing a wide spectrum of impairments across endocrine, skeletal, cardiovascular, immune, and psychological systems [1].

However, investigating RED‐S and its purported origin in LEA remains methodologically challenging. Most studies rely on estimations of energy availability based on self‐reported dietary intake and approximations of total energy expenditure, including exercise energy expenditure (EE) and non‐exercise activity thermogenesis (NEAT) [3]. Both components are prone to substantial variability and measurement error, limiting the precision of energy availability calculations and complicating causal attribution [3]. In this regard, Jeukendrup et al. [3] emphasize that many of the endocrine and metabolic alterations associated with RED‐S may not be solely attributable to LEA, but instead reflect the combined influence of additional stressors such as high training loads, psychological strain, inadequate sleep, and suboptimal macronutrient composition.

Understanding the short‐term physiological effects of LEA is particularly relevant given its high prevalence in various athletic contexts. In weight‐sensitive sports such as endurance, weight‐category, and aesthetic disciplines, athletes often adopt restrictive eating practices to achieve temporary weight loss [4, 5]. In addition, LEA can also arise unintentionally in endurance sports characterized by high training volumes, where the substantial increase in energy expenditure is not adequately matched by dietary intake [6]. Although these patterns are common, the repeated effects of short‐term LEA exposures on physiological homeostasis remain poorly understood.

From a mechanistic perspective, LEA can induce several hormonal and biochemical changes that reflect systemic attempts to conserve energy [7] including reductions in leptin, insulin, and insulin‐like growth factor 1 (IGF‐1), and triiodothyronine (T3), as well as alterations in reproductive hormones [8, 9] and changes in bone remodeling markers, such as Procollagen type 1 N‐terminal propeptide (P1NP) and C‐terminal telopeptide of type I collagen (βCTX‐1) [10]. Beyond these endocrine alterations, problematic LEA has been linked to shifts in iron metabolism (e.g., hepcidin, ferritin), lipid profile (HDL, LDL, triglycerides) [1], although findings are often inconsistent and influenced by study design and population characteristics. Therefore, the aim of this review was to systematically examine and map the effects of short‐term experimentally induced LEA on key biochemical responses in athletic populations.

## 2 Methods

This systematic review was conducted in accordance with the PRISMA (Preferred Reporting Items for Systematic Reviews and Meta‐Analyses) guidelines [11]. The systematic review was registered on the Open Science Framework (https://osf.io/rce84) on 9 November 2024.

### 2.1 Study Inclusion and Exclusion Criteria

The review parameters were established using the PICOS framework (Population, Intervention, Comparator, Outcomes, and Study design), which guided the inclusion criteria and overall structure of the review.

#### 2.1.1 Population

Eligible participants included male or female athletes from any type of sport discipline, including endurance, strength, or team‐based modalities. No restrictions were applied regarding training status, provided that participants could be classified between Tier 1 (recreationally active) and Tier 5 (world‐class elite) according to the framework proposed by McKay et al. [12] Sedentary individuals and clinical populations—including those with chronic diseases, obesity, eating disorders, or any other diagnosed condition known to alter energy metabolism or physiological responses—were also excluded.

#### 2.1.2 Intervention

Interventions involved experimental conditions of LEA lasting longer than 24 h, quantified as energy availability expressed in kcal·kg FFM^−1^·day^−1^. To be included, studies were required to explicitly report an EA or energy intake value equal to or below 30 kcal·kg FFM^−1^·day^−1^. Studies were excluded if LEA was inferred solely through relative reductions in habitual energy intake without reference to fat‐free mass.

#### 2.1.3 Comparator

Eligible studies were required to include a pre–post comparison of the intervention (LEA) within participants. Additionally, to ensure methodological rigor, only studies that incorporated both an LEA intervention group and a control condition (adequate energy availability) were included. This criterion was applied to minimize the risk that observed biochemical changes could be attributed to external factors rather than to the dietary manipulation itself.

#### 2.1.4 Outcomes

Eligible studies had to report at least one outcome related to the physiological effects of LEA. Outcomes were not pre‐selected a priori; all physiological and biochemical outcomes reported in the included studies were considered eligible for inclusion. For the purposes of synthesis, outcomes were grouped into the following domains:

- Bone metabolism biomarkers: βCTX‐1, P1NP, sclerostin, and osteocalcin.
- Calcium metabolism: Calcium, magnesium, phosphate, and parathyroid hormone (PTH).
- Energy and metabolic biomarkers: Leptin, cortisol, ghrelin, and IGF‐1.
- Inflammatory markers: Interleukin 6 (IL‐6) and C‐reactive protein (CRP).
- Iron metabolism: Ferritin, serum iron, hepcidin, hematocrit, hemoglobin, red blood cells, reticulocytes, transferrin, and transferrin saturation.
- Sex hormones: 17β‐estradiol, progesterone, follicle‐stimulating hormone (FSH), luteinizing hormone (LH), sex hormone‐binding globulin (SHBG), and testosterone.
- Thyroid function: T3 and thyroid‐stimulating hormone (TSH).

#### 2.1.5 Study Design

This review included original experimental studies, both randomized and non‐randomized, with either within‐subject (crossover) or between‐subject (parallel) designs. Observational studies, case reports, reviews, conference abstracts, and non‐peer‐reviewed publications were excluded. Only studies published in English in peer‐reviewed journals were considered for inclusion.

### 2.2 Information Sources and Search Strategy

The systematic electronic search was carried out in PubMed, Web of Science, and Scopus, and was conducted by IGC and PRP. The search strategy combined keywords across four conceptual blocks:

- *Nutritional intervention*

“Low energy availability” OR “energy availability” OR “reduced energy availability” OR “energy deficiency” OR “restricted energy”

- *Outcome*

“metabolism” OR “health” OR “outcome” OR “psycholog*” OR “physiolog*” OR “endocrine” OR “biomark*” OR “mark*” OR “biochemi*”

- *Population*

(“exercis*” OR “recreational*” OR “athlet*” OR “active” OR “player*” OR “sport*” OR “physically” OR “train*”)

- *NOT*

“review” OR “mouse” OR “animal*” OR “mice” OR “rats” OR “patholog*”.

The complete search strings for each database are provided in Supporting Information 1.

### 2.3 Selection of Studies

Three reviewers (IGC, NRP, and ABP) independently conducted the study selection process in two stages. In the first phase, titles and abstracts retrieved from the search were screened against the predefined inclusion and exclusion criteria. Articles that did not meet the eligibility requirements were excluded. In the second phase, full‐text articles were assessed to confirm their inclusion. Discrepancies between reviewers were discussed and resolved by consensus.

### 2.4 Data Extraction and Management

Data extraction was carried out by two team members (IGC and PRP) using a standardized template developed prior to the screening phase. The extracted data were then independently re‐checked by one of the original reviewers (IGC). Discrepancies were resolved by revisiting the original articles and discussing them among IGC, PRP, and ABP. If needed, a fourth team member (NRP) was consulted to reach consensus. In cases of incomplete or missing data, study authors were contacted directly to obtain the necessary information. In crossover studies with multiple LEA conditions, only one was included to avoid data duplication and ensure independence of observations. The selected group was the most relevant to the research question, usually the one with the lowest energy availability. Meta‐analysis could not be conducted for any of the variables due to an insufficient number of eligible intervention comparisons (*n* < 10) [13], which prevented obtaining robust pooled estimates.

### 2.5 Quality Assessment of Included Studies

The methodological quality of the included studies was assessed using the National Institutes of Health (NIH) Quality Assessment Tool for Before‐After (Pre‐Post) Studies With No Control Group. Although all studies included in this review applied a controlled intervention design—either randomized or non‐randomized, and many with crossover structures—the chosen tool was deemed appropriate because the outcomes of interest were primarily assessed based on within‐subject pre‐ to post‐intervention changes, rather than between‐group comparisons. Each item is rated as ‘yes’ (indicating low risk of bias), ‘no’ (high risk of bias), or ‘unclear’ (unclear risk of bias). Two independent reviewers (IGC and PRP) conducted the quality assessment. Disagreements were discussed until a consensus was reached. The results of the quality appraisal were considered in the interpretation of findings, although no studies were excluded based on their risk of bias scores.

## 3 Results

### 3.1 Literature Search

**FIGURE 1:**
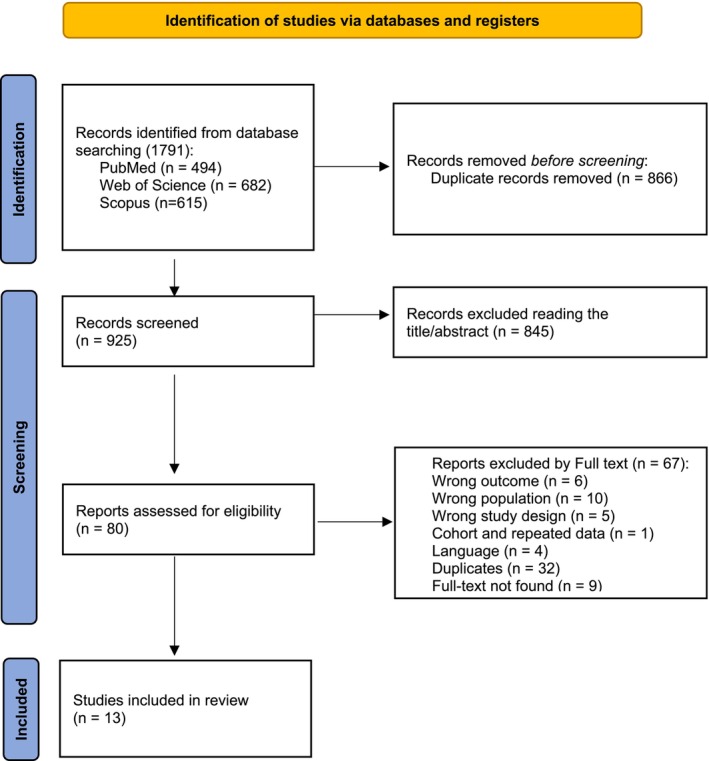
PRISMA flow diagram outlining the study selection process for the review.

The process of literature identification and study selection is summarized in Figure 1.

### 3.2 Study Characteristics

**FIGURE 2:**
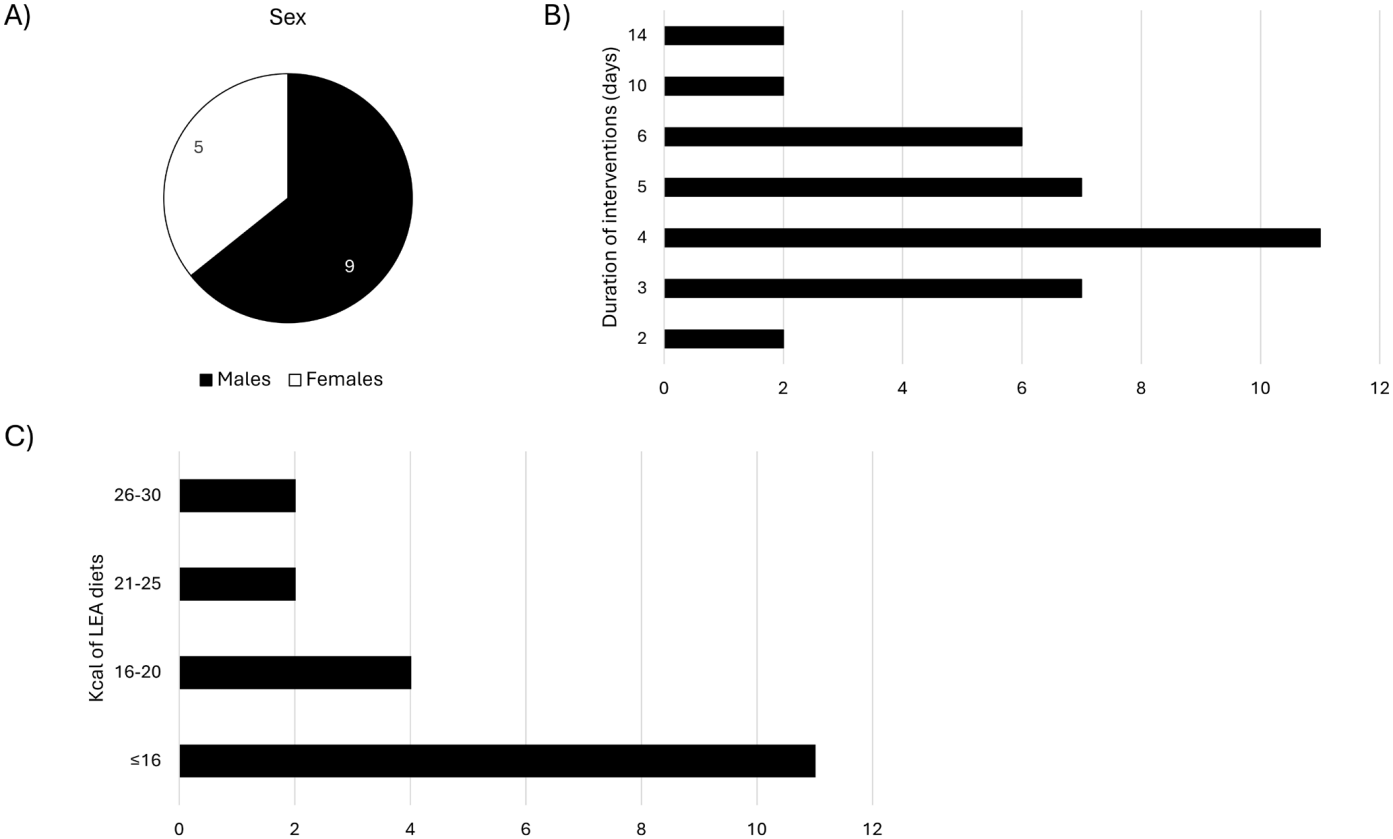
Characteristics of the intervention groups included in the review. (A) Sex distribution of participants in the intervention groups. (B) Duration of the LEA interventions (in days), ranging from 2 to 14 days. (C) Energy availability levels in the included interventions, expressed in kcal·kg^−1^ FFM·day^−1^. White bars represent LEA interventions, while black bars represent control interventions. LEA: Low energy availability.

A total of 13 studies [10, 14, 15, 16, 17, 18, 19, 20, 21, 22, 23, 24, 25], comprising 145 participants, were included in this review. Comprehensive characteristics of the included studies and participants are presented in Table 1, Table 2, and Figure 2. Nutritional characteristics of LEA interventions are shown in Table 3.

**TABLE 1:** Characteristics of participants and study design of the included studies. Data are presented as mean ± standard deviation. Training status was categorized according to descriptions in the original articles. The ‘Intervention’ column indicates the type of intervention conducted in each study.

ID	References	Interventions	*n*	Age (years)	Menstrual cycle phase	Training status	VO_2_max (ml · kg−1 · min−1)	Height (cm)	Weight (kg)	Outcomes
**Males**										
1	Fensham et al. [14]	LEA	10	30 ± 3		Elite male racewalkers	62.1 ± 6.1		67.8 ± 5.4	Bone: ↑βCTX‐1, ↓P1NP, ↔Gla‐OC, ↓Glu‐OC
2	Ishibashi et al. [22]	LEA	6	19.8 ± 0.4		Well‐trained long distance	67.9 ± 1.5	174 ± 1.2	59.9 ± 1.7	Inflammation: ↔IL‐6 Iron: ↑Ferritin ↔Hepcidin, ↑Fe
3	Koehler et al. [20]	LEA + Exercise	6	25.2 ± 1.0		Exercising	49.3 ± 2.4		79.7 ± 3.1	Energy: ↓Leptin, ↔Ghrelin, ↔IGF‐1 Thyroid: ↔T_3_ Sex: ↔T
4	Kojima et al. [18]	LEA	7	19.9 ± 1.1		Well‐trained athletes	67.5 ± 4.3	175.6 ± 4.7	61.4 ± 5.3	Energy: ↔IGF‐1 Sex: ↓T
5	Kojima et al. [19]	LEA	10	21.4 ± 0.6		Active	56.5 ± 1.3	170.4 ± 1.4	62.4 ± 1.5	Energy: ↓Leptin Sex: ↔T
6	McKay et al. [17]	LEA	10	30.0 ± 4.0		Elite race walkers	62.1 ± 6.1		67.8 ± 5.4	Energy: ↔Cortisol Inflammation: ↔CRP Iron: ↔Ferritin, ↔Hematocrit, ↔Hb, ↔Hepcidin, ↔Fe, ↔RBC, ↔Reticulocytes, ↔Transferrin, ↔TS Sex: ↓T
7	Murphy et al. [16]	LEA	7	23.9 ± 1.5		Trained	42.6 ± 2.4		86.9 ± 2.9	Bone: ↑βCTX‐1, ↓P1NP, ↔Sclerostin Energy: ↔IGF‐1, ↓Leptin
8	Papageorgiou et al. [10]	LEA	11	26 ± 5		Physically active	54.2 ± 5.3	178 ± 7	73.1 ± 8.0	Bone: ↔ βCTX‐1, ↔P1NP, ↔Sclerostin Calcium: ↔Ca, ↔Mg, ↔PO_4_, ↔PTH Energy: ↔Leptin, ↔IGF‐1 Sex: ↔E_2_ Thyroid↔T_3_
9	Sim et al. [25]	LEA	12	25.8 ± 3.8		Trained	52.2 ± 0.4	171.1 ± 4.8	61.2 ± 6.5	Bone: ↔βCTX‐1, ↔P1NP, ↔OC Energy: ↔Leptin, ↔IGF‐1 Sex: ↔E_2_, ↓T
**Females**										
10	Hutson et al. [23]	LEA	9	25.0 ± 3.5	Early follicular	Moderately/highly physically		165.6 ± 4.7	63.5 ± 6.6	Bone: ↑βCTX‐1, ↓P1NP Calcium: ↔Ca, ↔PO_4_, ↔Mg Sex: ↔E_2_ Thyroid: ↓T_3_
		LEA + Jump	10	19.0 ± 4.8	Early follicular	Moderately/highly physically		162.9 ± 7.2	59.0 ± 9.0	Bone: ↔βCTX‐1, ↓P1NP Calcium: ↔Ca, ↔PO_4_, ↔Mg Sex: ↔E_2_ Thyroid: ↔T_3_
11	Jeppesen et al. [21]	LEA	12	26.8 ± 3.4	Not reported	Trained	55.2 ± 5.1	169.8 ± 7.1	62.2 ± 8.8	Energy: ↑Cortisol Sex: ↔E_2_, ↔P_4_, ↔FSH, ↔LH, ↓T
12	Oxfeldt et al. [15]	LEA	14	26 ± 3	Early follicular and late follicular*	Trained	45.5 ± 3.2		64.3 ± 7.4	Energy: ↔Cortisol Sex: ↑SHBG, ↔T Thyroid: ↓T_3_, ↓TSH
8	Papageorgiou et al. [10]	LEA	11	26 ± 5	Early‐mid follicular	Physically active	47.9 ± 5.5	166 ± 5	59.7 ± 6.7	Bone: ↑βCTX‐1, ↓P1NP, ↔Sclerostin Calcium: ↔Ca, ↔Mg, ↔PO_4_, ↔PTH Energy: ↓Leptin, ↔IGF‐1 Sex: ↔E_2_ Thyroid: ↔T_3_
13	Papageorgiou et al. [14]	LEA + Exercise	10	24 ± 3	Early‐mid follicular	Recreationally active	48.1 ± 3.3	166 ± 5	61.1 ± 7.0	Bone: ↔βCTX‐1, ↔P1NP Calcium: ↔Calcium, ↔Mg, ↔PO_4_, ↔PTH Energy: ↓Leptin, ↓IGF‐1 Sex: ↔E_2_ Thyroid: ↔T_3_

Abbreviations: βCTX‐1, C‐terminal telopeptide of type I collagen; Ca, calcium; CRP, C‐reactive protein; E_2_, estradiol; Fe, serum iron; FSH, follicle‐stimulating hormone; Gla‐OC, undercarboxylated osteocalcin; Glu‐OC, γ‐carboxylated osteocalcin; Hb, Hemoglobin; IGF‐1, insulin‐like growth factor 1; IL‐6, interleukin 6; LEA, Low Energy Availability; LH, luteinizing hormone; Mg, magnesium; OC, osteocalcin; P1NP, procollagen type 1 N‐terminal propeptide; P_4_, progesterone; PO_4_, phosphate; PTH, parathyroid hormone; RBC, red blood cells; SHBG, sex hormone–binding globulin; T, testosterone; T_3_, triiodothyronine; TS, Transferrin saturation; TSH, thyroid‐stimulating hormone; VO_2_max, maximal oxygen uptake.

**TABLE 2:** Characteristics of the exercise interventions implemented in the studies included.

No	References	Group	Type of training	Frecuency	Volume (per day)	Intensity
1	Fensham et al. [14]	LEA	Racewalk	6 days	19.6 ± 2.7 km	
2	Hutson et al. [23]	LEA	None			
		LEA + Jump	Jumping exercise	3 days	4 sets x 5 reps	High impact
3	Ishibashi et al. [22]	LEA	Running	3 days	75 min	70% VO_2max_
4	Jeppesen et al. [21]	LEA				
5	Koehler et al. [20]	LEA + exercise	Cycle ergometer	6 days	Adjusted individually to expend 15 kcal · kg · FFM^−1^	60% VO_2peak_
6	Kojima et al. [18]	LEA	Running on a treadmill	3 days	75 min	~70% of VO_2max_ (15.2 ± 1.2 km·h^−1^)
7	Kojima et al. [19]	LEA	Running	2 days	60 min	70% of VO_2max_
8	McKay et al. [17]	LEA	Racewalk	6 days	19.6 ± 2.7 km	
9	Murphy et al. [16]	LEA	Cycle ergometer	7 days	Adjusted individually to expend 15 kcal · kg FFM^−1^ (115 ± 10 min)	60% of VO_2peak_ at 124 ± 12 W
10	Oxfeldt et al. [15]	LEA	Resistance training and cycle ergometer	3–4 days	Resistance: Upper and lower body—3 sets of 8–10 reps Endurance: 45–60 min	Resistance: An intensity of 12RM Endurance: 80% ventilatory threshold
11	Papageorgiou et al. [10]	LEA	Running on a treadmill	5 days	66 ± 4 min (14.8 ± 0.2 kcal · FFM^−1^ or 616 ± 74 kcal expended)	70% of VO_2peak_
12	Papageorgiou et al. [14]	LEA + exercise	Running on a treadmill	3 days	129 ± 10 min	70% of VO_2peak_
13	Sim et al. [25]	LEA	Running on a treadmill	4 days	Duration adjusted individually: 792 ± 82 kcal	65% VO_2max_

Abbreviations: FFM, fat‐free mass; kcal, kilocalories; LEA, low energy availability; reps, repetitions; RM, repetition maximum; VO_2_max, maximal oxygen uptake; VO_2_peak, peak oxygen uptake.

**TABLE 3:** Nutritional characteristics of studies included in this review.

	Fensham et al. [14]	Hutson et al. [23]	Ishibashi et al. [22]	Jeppesen et al. [21]	Koehler et al. [20]	Kojima et al. [18]	Kojima et al. [19]	McKay et al. [17]	Murphy et al. [16]	Oxfeldt et al. [15]	Papageorgiou et al.^a^[10]	Papageorgiou et al.^b^ [10]	Papageorgiou et al. [14]	Sim et al. [25]
Dietary duration (days)	6	3	4	14	4	4	2	6	5	10	5	5	3	4
Energy intake (kcal · day^−1^)	2335 ± 238	655 ± 36	2042 ± 75			2053 ± 170	1788 ± 53	2335 ± 238		1349 ± 164	1261 ± 125	1720 ± 235		1583 ± 165
Energy intake FFM^−1^ (kcal · kg^−1^· day^−1^)				22 ± 2	40			35 ± 3	30	25	30.5 ± 0.8	30 ± 0.2	45	
Energy availability·														
Kg FFM^−1^ (kcal·kg^−1^)	15 ± 2	15	17.3 ± 0.6		16.0 ± 0.5	17.9 ± 1.9	19.9 ± 0.3	15 ± 2	15	25	15	15	15	15
CHO (%)		50		45.6 ± 2.6	50–55	56.7 ± 4.7	51.6 ± 0.4	60			49 ± 8	48 ± 9	50	
Fat (%)		30		28.4 ± 1.5	30–35	23.0 ± 2.6	27.3 ± 0.2	15			33 ± 6	33 ± 7	30	
Pro (%)		20		26.0 ± 2.9	10–15	20.3 ± 3.4	21.1 ± 0.2	25			18 ± 5	19 ± 4	20	
CHO (g·day^−1^)	338 ± 33		288.1 ± 10.0	198 ± 33				338 ± 33		145 ± 20				198 ± 21
Fat (g·day^−1^)	40 ± 7		52.4 ± 4.1	55 ± 9				40 ± 7		39 ± 5				53 ± 5
Pro (g·day^−1^)	141 ± 14		100.5 ± 8.1	113 ± 21				141 ± 14		100 ± 11				79 ± 8
CHO· kg BW^−1^ (g·kg^−1^)	5.0 ± 0.4		4.8 ± 0.2*		3.1 ± 0.3*		3.71 ± 0.08*			2.5–4.0*				3.2*
Fat· kg BW^−1^ (g·kg^−1^)	0.6 ± 0.1				1.4 ± 0.2		0.87 ± 0.02							
Pro· kg BW^−1^ (g·kg^−1^)	2.1 ± 0.1				0.9 ± 0.1		1.51 ± 0.02							

Abbreviations: BW, body weight; CHO, carbohydrates; d, day; FFM, fat‐free mass; g, grams; kcal, kilocalories; kg, kilograms; Pro, protein.

^a^ Papageorgiou et al. [10]: Participants were females.

^b^ Papageorgiou et al. [10]: Participants were males.

* Data presented as mean ± SD, whereas values shown with a dash (−) represent ranges. CHO intake values below 5 g·kg^−1^·day^−1^ are indicated in bold and marked with an asterisk.

### 3.3 Methodological Quality

**FIGURE 3:**
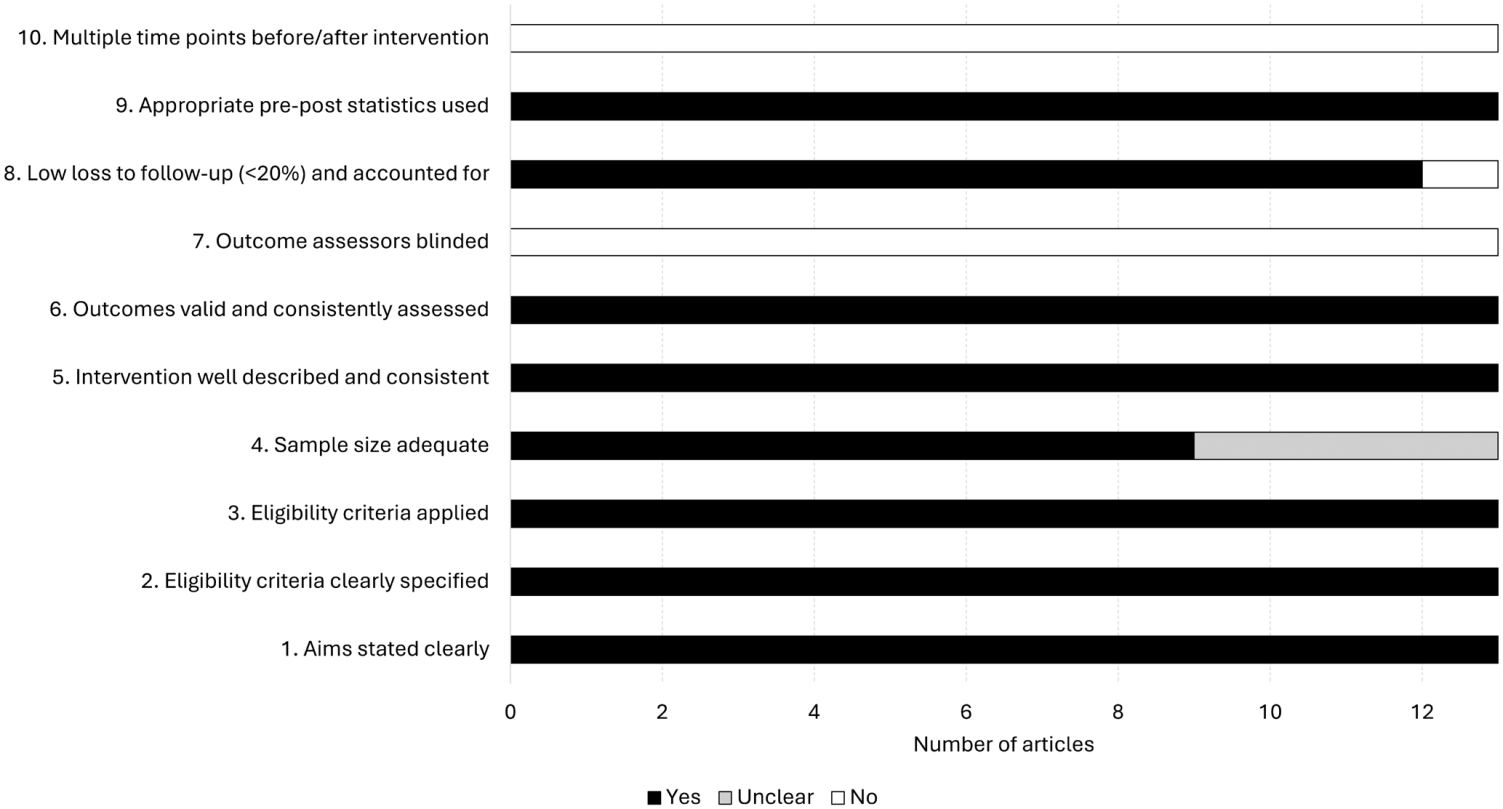
Overview of the National Institutes of Health (NIH) Quality Assessment Tool for Before‐After (Pre‐Post) Studies with No Control Group results.

Figure 3 presents an overview of the National Institutes of Health (NIH) Quality Assessment Tool for Before‐After (Pre‐Post) Studies with No Control Group results, highlighting the study design features with the highest risk of bias. Study scores ranged from 6 to 8 out of a possible 10 points (see Supporting Information 2), with a mean score of 7.6 ± 0.7.

#### 3.3.1 Bone Metabolism Biomarkers

Four [10, 16, 23, 24] out of eight intervention groups [10, 14, 16, 23, 24, 25] reported a significant increase in βCTX‐1 levels following LEA. Similarly, five [10, 16, 23, 24] out of eight [10, 14, 16, 23, 24, 25] showed a significant decrease in P1NP concentrations. Regarding osteocalcin, one [24] of the three [24, 25] interventions observed a significant reduction. Sclerostin was assessed in three interventions [10, 16], none of which reported significant changes post‐LEA.

#### 3.3.2 Calcium Metabolism

No significant post‐LEA changes were reported in any of the biomarkers related to calcium metabolism. This includes calcium (*n* = 5) [10, 14, 23], magnesium (*n* = 5) [10, 14, 23], phosphate (*n* = 5) [10, 14, 23], and PTH (*n* = 3) [10].

#### 3.3.3 Energy and Metabolic Biomarkers

Leptin concentrations were significantly reduced in five [10, 14, 16, 19, 20] out of eight [14, 16, 19, 20, 25] interventions and, among the three interventions [15, 17, 21] that assessed cortisol, one [21] reported a significant increase post‐LEA. IGF‐1 was significantly lower in 1 intervention [14] out of 8 [10, 14, 16, 18, 20, 25]. In contrast, no significant changes were observed in ghrelin (*n* = 1) [20].

#### 3.3.4 Inflammatory Markers

No significant effects of LEA were observed on inflammatory markers. IL‐6 (*n* = 1) [22] and CRP (*n* = 1) [17] were each assessed in separate studies, and neither showed significant changes following LEA exposure.

#### 3.3.5 Iron Metabolism

Among the biomarkers related to iron status, one out of two studies assessing ferritin reported a significant increase. Similarly, one of two studies showed a significant rise in serum iron post‐LEA. No significant changes were found in hepcidin (*n* = 2) [17, 22], hematocrit (*n* = 1) [17], hemoglobin (*n* = 1) [17], red blood cells (*n* = 1) [17], reticulocytes (*n* = 2) [17], transferrin (*n* = 1) [17], or transferrin saturation (*n* = 1) [17].

#### 3.3.6 Sex Hormones

17β‐estradiol was assessed in six studies [10, 14, 21, 23, 25], with none reporting significant changes. FSH, LH, and progesterone were each examined in one intervention [21], and no significant effects were observed between pre‐ and post‐LEA. In contrast, SHBG was evaluated in one study and showed a significant post‐LEA increase [15]. Testosterone was measured in eight interventions [15, 17, 18, 19, 20, 25], of which four reported significant reductions [17, 18, 21, 25].

#### 3.3.7 Thyroid Function

T3 was assessed in eight intervention groups [10, 14, 15, 20, 23], with two [15, 23] reporting a significant decrease post‐LEA. TSH was assessed in one study [15], which reported no significant change between pre‐ and post‐LEA intervention.

## 4 Discussion

This systematic review synthesized current evidence on the experimentally induced short‐term biochemical responses to LEA in athletes, focusing on biochemical markers related to bone, energy, iron, endocrine function, and inflammation.

Among the most consistent findings, approximately half of the interventions reported significant increases in βCTX‐1 and reductions in P1NP in response to LEA. These findings underscore the sensitivity of bone remodeling markers to short‐term LEA, even within a few days of LEA exposure. However, the magnitude of these responses appears to be influenced by several factors. For instance, Papageorgiou et al. [10] reported significant changes in female participants but not in males, suggesting potential sex‐specific susceptibility. Moreover, the type of LEA induction seems to influence the outcome: Papageorgiou et al. [14] showed that βCTX‐1 only increased when LEA was induced through caloric restriction alone, and not when exercise was included, pointing to a possible protective effect of physical activity. This aligns with findings by Hutson et al. [23], who observed that weight‐bearing exercise attenuated the alterations in bone turnover typically associated with LEA, further supporting the osteoprotective role of mechanical loading. Furthermore, dietary composition—particularly carbohydrate availability—may also influence bone metabolism independently of energy status [24]. These observations impair the interpretation of LEA effects and underscore the need for more controlled, mechanistic studies that isolate the impact of energy availability from other confounding variables. In contrast, markers related to calcium and phosphate metabolism showed no significant changes in any of the studies. This may suggest that homeostatic mechanisms tightly regulate these variables during short‐term LEA exposure, or that an adequate intake of these micronutrients may help preserve their physiological levels and functions even under conditions of energy deficiency.

Regarding metabolic and hormonal markers, consistent reductions were observed in leptin levels following LEA. Leptin, a hormone secreted by adipose tissue and strongly associated with fat mass [26], decreases rapidly during energy restriction, even before any measurable changes in body composition occur [27]. Although evidence remains limited, an interesting pattern emerged: Of the three studies that did not report significant changes in leptin, two were conducted in male participants and one in females, where LEA was induced primarily through increased exercise energy expenditure rather than dietary restriction. This observation suggests that both sex and exercise may influence leptin sensitivity, potentially reflecting a protective effect of male sex or of exercise itself. In contrast, in the present review, IGF‐1 levels remained unchanged across all eight interventions that assessed this marker, challenging its reliability as a short‐term indicator of LEA. IGF‐1 is a hepatic peptide hormone that mediates some of the anabolic effects of growth hormone (GH) [28]. In clinical contexts such as anorexia nervosa, females often exhibit elevated GH secretion alongside suppressed IGF‐1 levels, reflecting a state of hepatic GH resistance induced by LEA [7, 29]. Similarly, experimental studies in sedentary women undergoing a 5‐day dietary intervention have shown that energy availability at or below 30 kcal/kg of lean body mass per day can disrupt both GH secretion and IGF‐1 concentrations [30]. A potential explanation lies in the exercise and population characteristic: While the protocols included in this review all involved participants maintaining high‐intensity exercise during the LEA intervention, the studies by Loucks and Thuma [30] included sedentary participants and, although they incorporated exercise in the protocol, the absolute intensity and duration of the sessions (i.e., walking intermittently at ~70% VO_2_max) may not have been sufficient to elicit the same protective or anabolic adaptations. Therefore, it would be plausible that regular or more intense exercise may attenuate or counteract the IGF‐1 suppression typically observed in trained athletes, thereby preserving anabolic signaling during the short‐term LEA. In addition to these findings, only one study reported a significant increase in cortisol levels following LEA [21]. Cortisol plays a central role in energy mobilization and the physiological stress response, and its elevation is often considered an adaptive mechanism during energy deficiency [31]. However, the current evidence is too limited to determine whether cortisol is a reliable short‐term indicator of LEA. Moreover, cortisol concentrations can be influenced by numerous confounding factors—such as training load, overtraining, psychological stress, and the timing of sample collection [32]—making it difficult to isolate the specific effects of LEA. Therefore, further research is needed to clarify the role of cortisol in this context.

Similarly, there is currently insufficient evidence to determine whether short‐term LEA induces measurable changes in inflammatory markers. Only one study assessed IL‐6^22^, and another evaluated CRP [17], with neither reporting significant alterations post‐intervention. This limited evidence precludes any definitive conclusions. The absence of an inflammatory stimulus is also relevant when interpreting iron metabolism, given that IL‐6 upregulates hepcidin—an iron‐regulatory hormone that limits iron availability during inflammation [33]. In this review, although it was reported that increased ferritin and serum iron levels [17] post‐LEA in control and intervention groups, the majority of iron‐related parameters—including hepcidin, hemoglobin, red blood cells, reticulocytes, and transferrin—remained unchanged. However, it is important to note that markers of iron status, including hemoglobin, red blood cell count, and serum ferritin, typically respond over weeks rather than days [34]. Consequently, short‐term interventions may be insufficient to induce detectable changes in these markers; however, as previously noted, the limited available evidence precludes definitive conclusions.

Testosterone was the only sex hormone that demonstrated relatively consistent alterations in response to LEA, with 4 out of [17, 18, 21, 25] of 8 interventions reporting significant reductions across durations ranging from 4 to 14 days. These findings show that while reduced testosterone may be a consequence of short‐term LEA, the current evidence is insufficient to establish a clear dose–response relationship or temporal pattern. From a physiological perspective, testosterone suppression under LEA is likely mediated by the downregulation of the hypothalamic–pituitary–gonadal (HPG) axis [7, 8]. LEA can alter hypothalamic signaling—particularly the pulsatile release of gonadotropin‐releasing hormone (GnRH)—leading to reduced luteinizing hormone (LH) secretion and, consequently, decreased testicular testosterone production [7, 8]. However, the presence of mixed results across studies—likely influenced by methodological differences, participant characteristics, and dietary protocols—limits the ability to draw definitive conclusions regarding the consistency and magnitude of testosterone suppression in response to LEA. In contrast, no effects were observed for estradiol, progesterone, FSH, or LH. Despite hypoestrogenism being a key mechanism linking menstrual disturbances with bone and cardiovascular health in both the Female Athlete Triad [35] and RED‐S models [1, 7], current findings suggest that the suppressive effects of LEA on estradiol require longer exposure times. Therefore, although amenorrhea and hypoestrogenism are among the most visible signs of RED‐S [1, 7] and are frequently attributed to LEA, there is still no clear evidence regarding the specific magnitude or duration of LEA necessary to disrupt the hypothalamic–pituitary–ovarian axis in the short term. Moreover, assessing the effect of LEA on female sex hormones represents a methodological challenge, as these hormones fluctuate naturally across the menstrual cycle. Consequently, any changes observed could be partly explained by menstrual phase variation rather than LEA itself [36, 37]. In addition, these findings suggest that the alterations in bone remodeling markers—namely the increase in βCTX‐1 and the decrease in P1NP—in the short term are unlikely to be estrogen‐mediated, reinforcing the notion that LEA and hypoestrogenism exert both independent and combined effects on bone health over the long term [38].

Thyroid hormones, particularly T3, play a crucial role in regulating metabolism, growth, and reproductive function, and are known to respond adaptively to changes in energy availability. Under conditions of long‐term LEA, particularly in the clinic population with anorexia nervosa, suppression of T3 is considered a hallmark of the body's effort to reduce basal metabolic rate and conserve energy, often manifesting as a ‘sick euthyroid’ profile [39]. In athletic populations, consistently reduced T3 concentrations have been reported, whereas levels of T4 and TSH remained stable [40]. This is consistent with the identification of low total or free T3—whether subclinical or clinical—as a primary marker of RED‐S [1]. However, the present review found that T3 concentrations remained stable in most short‐term interventions. Only two out of eight studies reported significant reductions in T3 [15, 23], both conducted in female participants. This finding suggests potential sex‐specific sensitivity, with females showing more consistent hormonal adaptation to short‐term LEA [15, 23], supporting the hypothesis that men may be less vulnerable to thyroidal adaptation in the short term.

In summary, the differential sensitivity of physiological systems to short‐term LEA likely reflects the hierarchical nature of energy allocation in the human body. According to Loucks [41], to conserve energy for essential survival processes, the body undergoes metabolic and physiological adaptations, such as reduced reproductive function and compromised bone health, redirecting energy away from non‐essential functions like growth and maintenance functions such as tissue turnover, as well as reproductive development and function. These adjustments help the body achieve a new energy‐balanced state. In this context, bone remodeling markers (e.g., P1NP and βCTX‐1) may exhibit rapid fluctuations due to the high metabolic cost of the bone remodeling process, which can be transiently downregulated without immediately impacting other indicators of bone health, such as bone mineral density or parameters related to bone shape, size, and quality [42]. Similarly, hormones involved in metabolic regulation, such as leptin, exhibit quick responsiveness to shifts in energy balance due to their close association with energy intake and adiposity. In contrast, the reproductive and thyroid axes may require a more prolonged or severe energy deficit to exhibit measurable changes, as these systems are regulated by more complex neuroendocrine feedback loops and may be buffered against short‐term fluctuations to preserve reproductive potential and metabolic stability.

*Methodological considerations*.

From a methodological standpoint, a key limitation of the current body of literature is the heterogeneity of LEA protocols and macronutrient distributions across studies. Notably, many interventions achieved LEA by reducing CHO intake, often resulting in CHO availability below current sport nutrition recommendations for moderate exercise programs (< 5 g/kg/day) [43], particularly in weight‐sensitive disciplines. Our review revealed that several studies employed CHO intakes well below these thresholds (Table 3), while still meeting minimum protein intake guidelines (≥ 1.2 g·kg^−1^·day^−1^) [43]. This raises a major confounding issue: It is unclear whether observed physiological changes are solely due to LEA or also influenced by low CHO availability. Therefore, future studies should consider matched‐CHO control groups or designs that isolate the effects of macronutrient manipulation from those of energy availability. Additionally, micronutrient intake was not consistently reported across the included studies. Given that micronutrient availability may influence hematological and metabolic biomarkers, the absence of this information represents an additional limitation when interpreting blood‐based outcomes.

Additionally, the heterogeneity in study designs, populations (male vs. female, highly trained vs. recreational athletes), and outcome measures complicates direct comparisons and limits generalizability. Some biomarkers may also respond differently depending on sex, hormonal status, or the timing of sample collection relative to exercise or feeding. Furthermore, future research should prioritize sex‐specific investigations, as the physiological responses to LEA may differ between males and females in both nature and magnitude. Most current studies have small sample sizes and focus exclusively on one sex, limiting the ability to draw conclusions about sex‐specific differences. Given that hormonal regulation, bone metabolism, and metabolic adaptation are heavily influenced by sex hormones, it is plausible that LEA triggers distinct biochemical and endocrine responses in female vs. male athletes. Understanding these sex‐specific adaptations is essential to developing tailored prevention strategies and optimizing performance and health outcomes across athletic populations. To strengthen methodological rigor in studies involving female athletes, researchers should follow established recommendations [37], including accurate classification of hormonal status (e.g., naturally menstruating, eumenorrheic, oral contraceptive users) and clear reporting of menstrual cycle phase at data collection. This is particularly important as certain biomarkers may vary across menstrual cycle phases [44].

## 5 Conclusion

Short‐term experimentally induced LEA drives early alterations in key biochemical markers, particularly affecting bone remodeling markers (βCTX‐1, P1NP), leptin, and testosterone levels. These hormonal changes may represent short‐term, potentially reversible adaptations, with no clear evidence of clinically relevant consequences beyond the acute phase, while remaining potentially informative as biomarkers of under‐fueling during critical phases of athletic preparation. Other indicators, such as estradiol, IGF‐1, and T3, remained stable, suggesting that not all systems respond equally or as rapidly to energy restriction. The high heterogeneity in study designs, populations, and dietary protocols, especially regarding macronutrient distribution, limits definitive conclusions. Future research should address these gaps with more standardized and sex‐specific protocols to better understand the physiological consequences of short‐term LEA in athletes.

### 5.1 Perspectives

Accurate identification of LEA remains a major methodological challenge and is essential for the appropriate application of the RED‐S framework. Recent evidence indicates that LEA determination may be substantially influenced by both the quantification of energy intake, yielding systematically higher estimates when assessed using doubly labeled water compared with photographic or self‐reported methods [45], and the calculation of energy expenditure [3]. This has important implications for experimental research, as inaccurate estimates of energy availability may confound observed associations between LEA and biochemical outcomes. Consequently, the present review focused exclusively on experimentally controlled LEA conditions to minimize confounding related to energy intake assessment. Nevertheless, substantial challenges remain in the accurate quantification of exercise‐induced energy expenditure, which limits (or at least complicates) the establishment of fixed thresholds to define short‐term LEA effects.

From a clinical perspective, caution is required when interpreting the physiological relevance of acute biomarker perturbations observed in short‐term LEA models. Although RED‐S is fundamentally a condition arising from chronic LEA, short‐term experimentally induced LEA can elicit measurable, yet likely transient and reversible, changes in markers of bone remodeling, testosterone, and endocrine function. When followed by appropriate nutritional refeeding strategies or transient exercise stimuli, these short‐term perturbations are unlikely to result in clinically meaningful impairments, such as reductions in bone mineral density or major endocrine dysfunction. However, repeated or prolonged exposure to LEA, particularly when combined with low carbohydrate availability, high training loads, elevated stress, or other interacting factors, as proposed in the conceptual model by Jeukendrup et al. [3]., may lead to physiological consequences beyond the scope of the present review. Within this context, this review has aimed to identify the most sensitive biochemical markers responding to short‐term LEA, which may be useful in high training‐load scenarios where performance is prioritized, enabling early detection of under‐fueling and the optimization of nutritional strategies.

## Funding

The authors have nothing to report.

## Ethics Statement

The authors have nothing to report.

## Consent

The authors have nothing to report.

## Conflicts of Interest

The authors declare no conflicts of interest.

## Supporting information


**Supporting Information 1:** Detailed search strategy adapted for each database (PubMed, Web of Science, and Scopus), including the number of records retrieved from each database.

**Data S2:** Supporting Information.

**Data S3:** Supporting Information.

## Data Availability

Data sharing not applicable to this article as no datasets were generated or analysed during the current study.
