# Supplementary material for: Biochemical Responses to Experimentally Induced Short‐Term Low Energy Availability in Athletes: A Systematic Review
Source: Scand J Med Sci Sports. 2026 Mar 7;36(3):e70249. doi: 10.1111/sms.70249 (PMC12967264; doi:10.1111/sms.70249)
Supplement: Supplementary file 2 — Data S2: Supporting Information. [file SMS-36-e70249-s003.docx]

**PubMed**

((("Low energy availability"[Title/Abstract] OR "energy availability"[Title/Abstract] OR "reduced energy availability"[Title/Abstract] OR "energy deficiency"[Title/Abstract] OR "restricted energy"[Title/Abstract]) AND ("metabolism"[Title/Abstract] OR "health"[Title/Abstract] OR "outcome"[Title/Abstract] OR "psycholog*"[Title/Abstract] OR "percep*"[Title/Abstract] OR "physiolog*"[Title/Abstract] OR "endocrine"[Title/Abstract] OR "biomark*"[Title/Abstract] OR "mark*"[Title/Abstract] OR "biochemi*"[Title/Abstract])) AND (("exercis*"[Title/Abstract] OR "recreational*"[Title/Abstract] OR "athlet*"[Title/Abstract] OR "active"[Title/Abstract] OR "player*"[Title/Abstract] OR "sport*"[Title/Abstract] OR "physically"[Title/Abstract] OR "train*"[Title/Abstract]))) NOT ("review"[Title/Abstract] OR "mouse"[Title/Abstract] OR "animal*"[Title/Abstract] OR "mice"[Title/Abstract] OR "rats"[Title/Abstract] OR "patholog*"[Title/Abstract])

Results: 494

**Web of Science**

((TI=("low energy availability" OR "energy availability" OR "reduced energy availability" OR "energy deficiency" OR “restricted energy”)) OR AB=("low energy availability" OR "energy availability" OR "reduced energy availability" OR "energy deficiency" OR “restricted energy”)) AND ((TI=("metabolism" OR "health" OR "outcome" OR "psycholog*" OR "percep*" OR "physiolog*" OR "endocrine" OR "biomark*" )) OR AB=("metabolism" OR "health" OR "outcome" OR "psycholog*" OR "percep*" OR "physiolog*" OR "endocrine" OR "biomark*" OR "mark*" OR "biochemi*")) AND ((TI=("exercis*" OR “recreational*” OR "athlet*" OR "active" OR "player*" OR "sport*" OR "physically" OR "train*")) OR AB=("exercis*" OR “recreational*” OR "athlet*" OR "active" OR "player*" OR "sport*" OR "physically" OR "train*")) NOT ((TI=("review" OR "mouse" OR "animal*" OR "mice" OR "rats" OR "patholog*")) OR AB=("review" OR "mouse" OR "animal*" OR "mice" OR "rats" OR "patholog*"))

Results: 682

**Scopus**

TITLE-ABS ( "low energy availability" OR "energy availability" OR "reduced energy availability" OR "energy deficiency" OR "restricted energy" ) AND TITLE-ABS ( "metabolism" OR "health" OR "outcome" OR "psycholog*" OR "percep*" OR "physiolog*" OR "endocrine" OR "biomark*" OR "mark*" OR "biochemi*" ) AND TITLE-ABS ( "exercis*" OR "recreational*" OR "athlet*" OR "active" OR "player*" OR "sport*" OR "physically" OR "train*" ) AND NOT TITLE-ABS ( "review" OR "mouse" OR "animal*" OR "mice" OR "rats" OR "patholog*" )

Results: 615
