# Supplementary material for: Biochemical Responses to Experimentally Induced Short‐Term Low Energy Availability in Athletes: A Systematic Review
Source: Scand J Med Sci Sports. 2026 Mar 7;36(3):e70249. doi: 10.1111/sms.70249 (PMC12967264; doi:10.1111/sms.70249)
Supplement: Supplementary file 3 — Data S3: Supporting Information. [file SMS-36-e70249-s001.docx]

**Supp. Table 2.** Individual scores for each study using the National Institutes of Health (NIH) Quality Assessment Tool for Before-After (Pre-Post) Studies With No Control Group.

|  | *Fensham et al. (2022)* | *Hutson et al. (2023)* | *Ishibashi et al. (2020)* | *Jeppesen et al. (2024)* | *Koehler et al. (2016)* | *Kojima et al. (2020)* | *Kojima et al. (2022)* | *McKay et al. (2022)* | *Murphy et al. (2021)* | *Oxfeldt et al (2023)* | *Papageorgiou et al (2017)* | *Papageorgioua et al. (2018)* | *Sim et al. (2024)* |
| --- | --- | --- | --- | --- | --- | --- | --- | --- | --- | --- | --- | --- | --- |
| Was the study question or objective clearly stated? | Yes | Yes | Yes | Yes | Yes | Yes | Yes | Yes | Yes | Yes | Yes | Yes | Yes |
| Were eligibility/selection criteria for the study population prespecified and clearly described? | Yes | Yes | Yes | Yes | Yes | Yes | Yes | Yes | Yes | Yes | Yes | Yes | Yes |
| Were all eligible participants that met the prespecified entry criteria enrolled? | Yes | Yes | Yes | Yes | Yes | Yes | Yes | Yes | Yes | Yes | Yes | Yes | Yes |
| Was the sample size sufficiently large to provide confidence in the findings? | Yes | Yes | Unclear | Unclear | Yes | Yes | Yes | Unclear | Unclear | Yes | Yes | Yes | Yes |
| Was the test/service/intervention clearly described and delivered consistently across the study population? | Yes | Yes | Yes | Yes | Yes | Yes | Yes | Yes | Yes | Yes | Yes | Yes | Yes |
| Were the outcome measures prespecified, clearly defined, valid, reliable, and assessed consistently across all study participants? | Yes | Yes | Yes | Yes | Yes | Yes | Yes | Yes | Yes | Yes | Yes | Yes | Yes |
| Were the people assessing the outcomes blinded to the participants' exposures/interventions? | No | No | No | No | No | No | No | No | No | No | No | No | No |
| Was the loss to follow-up after baseline 20% or less? Were those lost to follow-up accounted for in the analysis? | Yes | Yes | Yes | Yes | Yes | Yes | Yes | Yes | No | Yes | Yes | Yes | Yes |
| Did the statistical methods examine changes in outcome measures from before to after the intervention? Were statistical tests done that provided p values for the pre-to-post changes? | Yes | Yes | Yes | Yes | Yes | Yes | Yes | Yes | Yes | Yes | Yes | Yes | Yes |
| Were outcome measures of interest taken multiple times before the intervention and multiple times after the intervention (i.e., did they use an interrupted time-series design)? | No | No | No | No | No | No | No | No | No | No | No | No | No |
|  |  |  |  |  |  |  |  |  |  |  |  |  |  |
| Total "Yes" | 8 | 8 | 7 | 7 | 8 | 8 | 8 | 7 | 6 | 8 | 8 | 8 | 8 |
| Total "No" | 2 | 2 | 2 | 2 | 2 | 2 | 2 | 2 | 3 | 2 | 2 | 2 | 2 |
| Total "Unclear" | 0 | 0 | 1 | 1 | 0 | 0 | 0 | 1 | 1 | 0 | 0 | 0 | 0 |
